# By Toutatis! Trainee Teachers’ Motivation When Using Comics to Learn History

**DOI:** 10.3389/fpsyg.2021.778792

**Published:** 2021-10-27

**Authors:** Juan Ramón Moreno-Vera, Santiago Ponsoda-López de Atalaya, Rubén Blanes-Mora

**Affiliations:** ^1^Faculty of Education, University of Murcia, Murcia, Spain; ^2^Faculty of Education, University of Alicante, Alicante, Spain

**Keywords:** social sciences, teaching-learning, historical thinking, motivation, comic

## Abstract

The main goal of this research was to analyse the perception of trainee primary education teachers regarding motivation when using comics as a resource to teach and learn history. To achieve this objective, a history education programme was designed based on the use of comics and the outcomes evaluated *via* a mixed qualitative-quantitative post-test questionnaire (Likert scale 1–5). Two hundred twenty-one trainee primary teachers from the University of Alicante, Spain participated in the study during the 2020/2021 academic year. Data were collected using the IBM SPSS v.24 statistical package and AQUAD 7. The results showed that the majority of future teachers felt highly motivated when using comic resources to learn history instead of textbooks (90.5% of participants); trainee teachers recognise that the use of comics improves their capacity to be more creative and that they feel able to design and use their own comic resources to teach history in the future.

## Introduction

Nowadays, the educational resources at the disposal of teachers are not only more numerous and diverse than a few years ago, but they also require a greater degree of knowledge when they are confined to different subjects or heterogeneous nature, enriching a little more one of the most important professions in our society. In the case of history teaching, the methodological revolution which has taken place over the past two decades has been unstoppable and raises the issue of the total renovation of the teaching profession (Boerman-Cornell, 2015; Souto and Martínez, 2016). This paper addresses the use of comics, and in some cases graphic novels (Montenegro, 2012; Sebastián-Faubel, 2016; Williams, 2008), as an educational resource on the rise which aims to facilitate history learning in a motivating way, encouraging critical thinking and, in turn, stimulating students’ creativity.

For many of us, brought up in a society in which the mass media are ever-present, cartoon strips and comics have formed part of what could be referred to as our sentimental upbringing (Gubern, 1977). It is true to state that never before have so many graphic novels been produced and read as is the case today. Thus, this manifestation requires considered reflection and, in our opinion, a suitable educational context in which these resources can be implemented in an effective manner. In this way, the educational potential which comics put at our disposal can be prioritised. If a correct reading is given, by stimulating observation, reading, analysing and interpreting a series of actions reflected in the cartoons, it is possible to consolidate and acquire a historical approach capable of handling and distinguishing between fiction, truth and reality (Lluch-Prats et al., 2016; Sebastián-Faubel, 2016).

In the words of McCloud (1993, p. 9), comics are juxtaposed illustrations and other images in deliberate sequences which aim to transmit information or obtain an aesthetic response from the reader. Gubern (1977), one of the sharpest analysts of the popular and visual culture of our times, characterised comics as a sequence of consecutive images which articulate a narrative in which at least one stable character and an integration of the text in the image can be found throughout the series. Therefore, the most imperative characteristic is the capacity of the comic or graphic novel to naturally integrate iconic and verbal language (Arango, 2012).

If these classical definitions, used in their time to characterise a society ever more focused on aesthetics (as predicted by Berger, 1990), are taken as a starting point, education cannot overlook one of the most important cultural manifestations of the past decades. In other words, it seems to be the ideal moment to incorporate these forms of mass media, along with others such as cinema and photography, into the classroom, adapting them to educational projects resulting from discussion and intellectual debate.

If, as stated by Gubern (1977), ours is a culture of the printed image for the masses, history teaching should provide itself with valid tools for this culture, not so much to ignore what has been done or taught to date, but to offer a re-reading or an alternative version of history (Arango, 2012; Steiner et al., 2014). In the words of Saitua (2018a), history teachers should provide their students with materials and training resources which foster a clear understanding of complex historical processes, enable them to identify historical causality and to develop the skills associated with argumentation. Consequently, comics, as stated by many different authors (Williams, 2008; Aguilera, 2011; Blay, 2015; Boerman-Cornell, 2015; Souto and Martínez, 2016), could be an ideal resource for history teaching and the development of historical thinking (Ortega-Sánchez and Pagès, 2017) due to their recreational and motivational character, plot clarity and multi-modal nature. For Zagkotas (2019) and Sebastián-Faubel (2016), the narrative formula of comics, an element which should, no doubt, be borne in mind, is another way of narrating the past which is closely linked with the narrative discourse of the historian. As the student is presented with the world *via* an imaginary lens, he/she is encouraged to seek information in an autonomous manner, observing the necessity to confront and contrast the documentary sources, thus fostering discussion and interpretation of the narration, leading to an attitude which is surely more positive concerning the place of history in his/her education.

In close relation to the concepts and ideas described here, Saitua (2018b) points out an element which is fundamental for our interests: reading a comic can enable the student to deploy the necessary mental frameworks for historical thinking. Without doubt, this is an important step in considering this means of expression as a first-order tool in the teaching of history, as is stated in the studies by Aguilera (2011), Boerman-Cornell (2015) and Souto and Martínez (2016). These authors stress the importance of the identification, contextualisation and corroboration of all of the historical elements which appear in the comic, interpreting them *via* a critical, efficient reading, a characteristic of history teaching itself. In this regard, Arango (2012), Saitua (2018a) and Blay (2015) state that this reading, formulated within an educational project which takes in some of the issues formulated above, not only helps to promote critical thinking among students, but also imagination and creativity. These authors consider that this stimulus, which promotes and encourages imagination, also takes place on what is specifically known as “historical imagination,” defined by Lowenthal (1985, p. 213) as “collective memory is constructed through shared images.” Indeed, the comic strips which present certain historical contents do not restrict themselves exclusively to returning to these shared mental images and reproducing them, as was the case with the well-known graphic novel by Art Spiegelman, *Maus: A Survivor’s Tale* (1992). But rather, they transform and insert new images into this ensemble, a fundamental aspect when their use is contemplated in the education of students who are in the process of forming their own historical imagination (Arango, 2012; Lluch-Prats et al., 2016).

Among the main advantages of using comics to teach history, it can be highlighted that not only must the student adopt a role as a researcher–reader, as mentioned by many authors (Aguilera, 2011; Montenegro, 2012; Souto and Martínez, 2016; Zagkotas, 2019), evaluating sources, interpreting information and considering different perspectives, but also different key concepts can be worked on, such as identity-otherness, multiple causality, change and continuity, diversity, inequality and conflict, according to the pattern proposed by Sebastián-Faubel (2016). Thus, comics, used as an educational resource, put into images what textbooks explain in words, fostering students’ creativity and increasing their interest in what is taught, as stated by Blay (2015). This is yet another incentive for the use of new methodologies in history teaching as it becomes possible for the teacher to catch his/her students’ attention thanks to the complementarity between what is iconic and what is written, as the task of opening the eyes of the reader is facilitated (Arango, 2012; Boerman-Cornell, 2015).

Ultimately, the challenge with which we are confronted when considering the suitability of the use of comics in the history classroom resides in the capacity of teachers to select which comics are to be used in class, what prior information to offer to students and, last but not least, what type of choice is sought from the reading and analysis of this educational resource (Montenegro, 2012; Steiner et al., 2014; Lluch-Prats et al., 2016). In brief, comics are a cultural product generated in certain specific social and productive conditions (Arango, 2012). Therefore, it is the teacher who is responsible for incorporating them into a broader ensemble of strategies with the aim of substantially transforming the teaching of history (Aguilera, 2011; Blay, 2015).

In this regard, the main aspects which make comics a resource of great educational potential lie in their capacity to motivate (Altarriba, 2003) and in the fact that they are considered to be a transversal and multidisciplinary tool which can facilitate integral learning (Jiménez García et al., 2019). Likewise, Barrero (2002), cited by Jiménez García (2020), points out certain reasons for the benefits of using comics in language teaching, which can, in our opinion, also be applied to other subjects, such as their narrative and visual aspects, the fostering of creativity and the capacity for reflection on cultural and social reality. To all of this can be added the fact that comics enable the introduction of specific contents for different subjects (Pons, 2017).

Along these lines, it can be stated that comics make it possible to work on values and beliefs (Onieva, 2015), reflect on the world around us and favour research (Guzmán, 2011). They are, therefore, an ideal resource for teaching and learning history as their use in the classroom is related, to a great degree, with the objectives and intentions of teachers (Gómez-Trigueros and Ruiz-Bañuls, 2019).

Taking into account the aforementioned statement, comics can be used in history classes in many different ways. First of all, comics can be used as a historical source to address the study and knowledge of the society which produces them (Gual Boronat, 2011, 2013; Sola Morales and Barroso Peña, 2014; Calviño Freire, 2018), as they can show features which reflect the moment of time in which they were created (Jiménez García, 2020). Secondly, another way of implementing the use of comics in the classroom is *via* the design of educational activities based on the contents of a published comic, making it possible to use works which address the specific period being studied or to be studied in class, thus transforming the comic into a tool enabling the study of a specific historical reality *via* argumentation (Saitua, 2018b; Fernández de Arriba, 2019). A third approach to working on historical contents based on comics is the creation of comics by teachers and students. This creation can be by hand or *via* the use of any of the different digital applications available on the Internet. Independently of the tool used to create them, in the case of teachers, the creation and use of comics in the classroom makes it possible to present contents in a different way which can be adapted to both the learning contents and objectives. As far as the creation of comics by students is concerned, this can be the final result of research in the style of a historical narrative.

## Materials and Methods

### Objectives

The main objective of this research was to analyse the motivation of future primary education teachers when using comics as an educational resource in the teaching of history. In order to achieve this objective, three specific objectives (SO) were proposed:

- SO 1: To analyse the elements of motivation that trainee teachers have when using comics as an educational resource in the teaching of history.
- SO 2: To study the positive aspects which future teachers consider when creating their own comics as an educational resource.
- SO 3: To establish the limitations and difficulties of the creation of comics for the teaching of history according to the trainee teachers.

This research is based on the assumption that, in general, the future primary education teachers have not worked with comics as a resource for learning history before and that, therefore, they have not considered comics as a future educational resource which can help them to improve their social sciences classes. Therefore, the following research questions have been proposed as a result of this work: Do the future teachers consider that comics are a motivating resource for history classes? What are their perceptions regarding motivation for learning social sciences?

### Participants and Context

This research was carried out on an intentional sample as its participants were, in all cases, trainee teachers from the third year of a degree in primary education at the University of Alicante, Spain.

The sample consisted of 221 future primary education teachers (*n*=221) taking the subject entitled “Didactics of the Social Sciences: History” in which the training programme took place.

As far as the characteristics of the participants are concerned, there was a total of 63 men (28.5%) and 158 women (71.4%) with an average age of 22.4years.

### An Educational Programme Based on Comics to Learn History

The ultimate objective of the educational programme developed was for students to learn to develop their own educational resources using comics as a tool for the teaching and learning of historical contents. For this purpose, first of all, the educational possibilities of comics were introduced.

Thus, in the first phase, an activity was proposed based on a series of cartoons from the comic *Asterix and the Chariot Race* (Gosciny et al., 2017a,b). Several questions were posed with the ultimate aim of working on a specific issue: the importance of roads for the creation and consolidation of the Roman Empire. This activity served as an example for the next educational proposal to be developed, which was based on the creation of a comic by the students. Each student was required to create a comic using a digital application (*Pixton* or *Storyboard*) focusing on a historical episode, character or event.

Once the comic had been created, the next step was to design a series of related activities to work on the contents addressed following the example of the first activity.

### Instrument Design, Validation and Research Process

The research tool was designed *ad hoc* for this study, although it was based on similar tools which have already been validated and published. Like the tool designed by Gómez-Carrasco et al. (2020), its aim was to reveal students’ motivation concerning the teaching and learning of the social sciences.

In order to carry out the research, a mixed quantitative and qualitative questionnaire was designed (Supplementary Material) with 10 closed numerical statements on a Likert scale (1–5) in ascending level of agreement and two items of an open qualitative nature in which the trainee teachers expressed the main positive and negative aspects of the educational programme.

The items analysed were:

- Item 1. The way the activity was presented and worked on has motivated me to learn more about history.
- Item 2. The way the activity was presented and worked on has motivated me to learn more about the use of comics as a resource for teaching and learning about history.
- Item 3. The activity has improved my motivation to make more of an effort in this subject.
- Item 4. The activity has improved my motivation to achieve better grades.
- Item 5. The activity has motived me because it has enabled me to contribute my knowledge.
- Item 6. The activity has motived me because it has enabled me to contribute my creative skills.
- Item 7. The activity has motived me because we have used resources which are different to those normally used.
- Item 8. The activity has motived me because I have been able to be responsible for my own learning.
- Item 9. The activity has motived me because it has taught me to design my own contents and activities via comics.
- Item 10. The activity has motived me because I consider it to be useful for my future career.
- Item 11 (open). State and explain the positive aspects of the creation of comics for the teaching and learning of history in primary education.
- Item 12 (open). State and explain the negative aspects of the creation of comics for the teaching and learning of history in primary education.

In order to validate the reliability of the construct, an internal consistency analysis (Supplementary Material) was carried out on the total of the numerical scale (*n*=10 items) which revealed a Cronbach’s alpha value of 0.902 (0.9>1, representing an extremely high degree of reliability according to Oviedo and Campo-Arias, 2005) and a value of 0.873 with the Guttman split-half coefficient (Supplementary Material), thereby demonstrating a high degree of internal reliability of the items on the scale according to other research in the field of social science education, such as that carried out by Gestsdóttir et al. (2018), Gómez-Carrasco et al. (2019) and Moreno-Vera et al. (2020).

Furthermore, as it is a mixed quantitative-qualitative questionnaire, the Friedman test was employed to establish whether there is dependence between the quantitative items and if these affect the qualitative items (Supplementary Material). In this case, the values of the Chi-square test vary between a low of 69.2 in item 4 to 264.6 for item 7, all of which are far removed from 0 (>0.05), which, according to Satorra and Bentler (2010) and Sharpe (2015), indicates that there is discrepancy between the variables and that, therefore, they are not dependent. This bestows a positive reliability value on the qualitative research, as occurs in other studies on the teaching of the social sciences, such as Moreno-Vera et al. (2020).

Lastly, as far as the research procedure is concerned, it must be pointed out that participation in the research was voluntary and subject to ethical guidelines, and the trainee teachers gave informed consent for their participation. Secondly, both the educational programme and the later test were applied in class time by professionals trained for that purpose. Thirdly, the analysis of the research results was carried out using IBM SPSS v. 24 software for the analysis of the descriptive statistics of the questionnaire, whereas the qualitative analysis was carried out with the AQUAD 7 program (Huber, 2013), establishing the variables based on the narratives of the trainee teachers.

## Results

First of all, as far as the analysis of the descriptive statistics is concerned, it must be stated that the analysis of the means, medians and SD (Supplementary Material) of the 10 quantitative items of the questionnaire has been extremely positive. The item with the lowest mean score is item 4 with 4.0 out of 5, which, in itself, represents a positive result. Thus, the mean values oscillate between 4.0 for item 4 (The activity has improved my motivation to achieve better grades) and 4.6 for item 7 (The activity has motived me because we have used resources which are different to those normally used).

In relation to the median, all of the items are situated between 4 and 5. However, in terms of the SD, all of the items analysed present a value of <1, thus representing a positive detail as no item has a great degree of variability. Item 7 (The activity has motived me because we have used resources which are different to those normally used) presents the greatest homogeneity of responses with the SD below 0.6, whereas item 5 (The activity has motived me because it has enabled me to contribute my knowledge) is that which presents the greatest variability with 0.9, which, in spite of everything, is a very positive piece of information.

### Motivation of Future Teachers When Using Comics as a Resource to Teach and Learn History

The statistical and descriptive analysis (Supplementary Material) of the items reveals extremely positive results concerning the perception of trainee primary education teachers regarding the use of comics as an educational resource for the learning of history.

Item 1 (The way the activity was presented and worked on has motivated me to learn more about history; Table 1) has a mean score of 4.3 out of 5, this score being the most represented with 52.5% of the students feeling high, motivated by comics as they enable them to obtain more conceptual contents on the subject of history. If the accumulated percentages of values 1, 2 and 3 are added up, it can be noted that only 14.5% of trainee teachers consider that the use of comics is not motivating in terms of learning about history.

**Table 1:** The way the activity was presented and worked on has motivated me to know more about history.

VAR00001				
Valid	Frequency	Percentage	Valid percentage	Cumulative percentage
1.00	2	0.9	0.9	0.9
2.00	1	0.5	0.5	1.4
3.00	29	13.1	13.1	14.5
4.00	73	33.0	33.0	47.5
5.00	116	52.5	52.5	100.0
Total	221	100.0	100.0	

Item 2 (The way the activity was presented and worked on has motivated me to learn more about the use of comics as a resource for teaching and learning about history; Table 2) has a positive mean score of 4.4. In this case, 89.2% of the trainee teachers responded that the activity motivated them to discover more about comics as a resource if the percentages of those who are “in agreement” and “totally in agreement” are taken into account. Only 7.3% of the students did not agree that the activity had motivated them to know more about the use of comics in the teaching of history.

**Table 2:** The way the activity was presented and worked on has motivated me to know more about the use of comics as a resource for teaching and learning about history.

VAR00002				
Valid	Frequency	Percentage	Valid percentage	Cumulative percentage
1.00	1	0.5	0.5	0.5
2.00	4	1.8	1.8	2.3
3.00	19	8.6	8.6	10.9
4.00	76	34.4	34.4	45.2
5.00	121	54.8	54.8	100.0
Total	221	100.0	100.0	

As for the statistical and descriptive analysis of item 3 (The activity has improved my motivation to make more of an effort in this subject; Table 3), the majority of the future teachers stated that they were in agreement. The mean for this item was 4.1 out of 5 and it is worthy of note that none of the participants stated that they were “totally in disagreement” with this statement and only 2.3% were in disagreement. On the other hand, the most commonly selected option was “in agreement” with 44.3% of the sample, whereas 38% of the participants were “totally in agreement.”

**Table 3:** Activity has improved my motivation to make more effort in the subject.

VAR00003				
Valid	Frequency	Percentage	Valid percentage	Cumulative percentage
2.00	5	2.3	2.3	2.3
3.00	34	15.4	15.4	17.6
4.00	98	44.3	44.3	62.0
5.00	84	38.0	38.0	100.0
Total	221	100.0	100.0	

Item 4 (The activity has improved my motivation to achieve better grades; Table 4), as explained above, is the item with the lowest mean value (4.0) which, even so, is an extremely positive result. As far as the percentage is concerned, it is worthy of note that there were no students who were “totally in disagreement” and only 2.7% were “in disagreement.” It is interesting to note how, in this item, 26.7% of the participants were neither in agreement nor disagreement, which demonstrates certain doubts when responding. This leads us to consider that improving grades and marks is not such an important issue for future teachers. On the other hand, those participants who do indeed feel motivated to obtain better grades represent similar values with 39.4% of the sample being “in agreement” and 33.9% “totally in agreement.”

**Table 4:** Activity has improved my motivation to achieve better grades.

VAR00004				
Valid	Frequency	Percentage	Valid percentage	Cumulative percentage
2.00	6	2.7	2.7	2.7
3.00	53	24.0	24.0	26.7
4.00	87	39.4	39.4	66.1
5.00	75	33.9	33.9	100.0
Total	221	100.0	100.0	

As for item 5 (The activity has motived me because it has enabled me to contribute my knowledge; Table 5), the mean score is 4.2 with 79.2% of the future teachers declaring that they were “in agreement” or “totally in agreement” that using comics as a teaching resource motivates them to obtain greater conceptual contents of history. In this case, only 5.9% of the participants were “in disagreement” with this statement.

**Table 5:** Activity has motivated me because it has enabled me to contribute my knowledge.

VAR00005				
Valid	Frequency	Percentage	Valid percentage	Cumulative percentage
1.00	1	0.5	0.5	0.5
2.00	11	5.0	5.0	5.4
3.00	34	15.4	15.4	20.8
4.00	69	31.2	31.2	52.0
5.00	106	48.0	48.0	100.0
Total	221	100.0	100.0	

Item 6 (The activity has motived me because it has enabled me to contribute my creative skills; Table 6) has a very positive mean score of 4.4. Only 2.8% of the trainee teachers are “in disagreement,” while the percentage of those in doubt is relatively low at 10.4%. As regards the motivation which they have felt due to being able to develop aspects of their own creativity *via* the activity with comics, the vast majority (89.6%) are “in agreement” that this creative aspect motivates them. Indeed, 58.8% of the participants claim to be “totally in agreement” with this point.

**Table 6:** Activity has motivated me because it has enabled me to contribute my creative skills.

VAR00006				
Valid	Frequency	Percentage	Valid percentage	Cumulative percentage
1.00	1	0.5	0.5	0.5
2.00	4	1.8	1.8	2.3
3.00	18	8.1	8.1	10.4
4.00	68	30.8	30.8	41.2
5.00	130	58.8	58.8	100.0
Total	221	100.0	100.0	

As stated above, the statistical analysis of item 7 (The activity has motived me because we have used resources which are different to those normally used; Table 7) has the highest mean score with 4.6 out of 5. It is noteworthy in this case that 71% of the future teachers are “totally in agreement” that using teaching resources which are different to those normally used proves to be a motivating factor. Furthermore, including the 19.5% who were “in agreement,” the positive percentage for this item increases to 90.5%, the highest of all the items. This indicates that the trainee teachers value positively the use of resources such as comics as it enables them to introduce resources other than the textbook (still the most commonly used resource) into their history classes, thereby offering the possibility of working on history *via* active learning methodologies and preventing students from playing a passive role in the classroom.

**Table 7:** Activity has motived me because we have used resources which are different to those normally used.

VAR00007				
Valid	Frequency	Percentage	Valid percentage	Cumulative percentage
2.00	3	1.4	1.4	1.4
3.00	18	8.1	8.1	9.5
4.00	43	19.5	19.5	29.0
5.00	157	71.0	71.0	100.0
Total	221	100.0	100.0	

When examining the analysis of item 8 (The activity has motived me because I have been able to be responsible for my own learning; Table 8), a high mean score of 4.3 out of 5 can be observed. 84.1% of the trainee primary teachers are “in agreement” or “totally in agreement” with the idea that playing an active role within the teaching and learning process is, in their opinion, a motivating factor. This result is related with the previous item and confirms that students feel more motivated in history classes when they are the ones to carry out activities relating to the application of knowledge rather than merely receiving the lesson in a passive way.

**Table 8:** Activity has motived me because I have been able to be responsible for my own learning.

VAR00008				
Valid	Frequency	Percentage	Valid percentage	Cumulative percentage
1.00	1	0.5	0.5	0.5
2.00	5	2.3	2.3	2.7
3.00	29	13.1	13.1	15.8
4.00	77	34.8	34.8	50.7
5.00	109	49.3	49.3	100.0
Total	221	100.0	100.0	

Item 9 (The activity has motived me because it has taught me to design my own contents and activities *via* comics; Table 9) also presents a positive mean score of 4.3. In this case, the trainee teachers also consider that the training programme working on history *via* comics motivated them as it enabled them to design their own comics and materials to use in the future with primary schoolchildren. 52.5% of the participants were “totally in agreement” and 36.7% were “in agreement.” Only 2.7% of the participants stated that they did not feel motivated designing and creating their own teaching materials in the format of a comic.

**Table 9:** Activity has motived me because it has taught me to design my own contents and activities *via* comics.

VAR00009				
Valid	Frequency	Percentage	Valid percentage	Cumulative percentage
2.00	6	2.7	2.7	2.7
3.00	18	8.1	8.1	10.9
4.00	81	36.7	36.7	47.5
5.00	116	52.5	52.5	100.0
Total	221	100.0	100.0	

Last of all, item 10 (The activity has motived me because I consider it to be useful for my future career; Table 10) obtained an extremely high mean score of 4.5 out of 5. What is more, 90.1% of the sample (the second highest percentage of the questionnaire) were “in agreement” with the idea that learning about history *via* comics will be useful in their future teaching careers in primary education. Indeed, the lowest value of those participants who were “totally in disagreement” of the whole questionnaire can be observed in this item (only 1.4% of the participants).

**Table 10:** Activity has motived me because I consider it to be useful for my future career.

VAR00010				
Valid	Frequency	Percentage	Valid percentage	Cumulative percentage
1.00	1	0.5	0.5	0.5
2.00	1	0.5	0.5	0.9
3.00	20	9.0	9.0	10.0
4.00	53	24.0	24.0	33.9
5.00	146	66.1	66.1	100.0
Total	221	100.0	100.0	

### Positive Perceptions on Creativity When Using Comics as a Resource for Teaching History

Item 11 of the questionnaire was an open question aimed at revealing the positive aspects which future teachers perceive regarding the use of comics as a resource for teaching and learning history in primary education (Table 11).

**Table 11:** Positive aspects regarding the use of comics as a resource for learning history.

Variable	Frequency	%
Favours understanding of contents	73	12.6
Motivation	72	12.4
Encourages creativity	69	11.9
Visual resource facilitates understanding	46	7.9
Entertaining resource	45	7.7
Innovative resource/innovation	37	6.4
Makes it possible to adapt contents	28	4.8
Favours autonomous and active learning	28	4.8
Makes it possible to work on basic competences	27	4.6
Makes it possible to learn in a different way	26	4.5
Favours working with competences associated with the development of historical thinking	23	4
Arouses students’ curiosity/interest/attention	19	3.3
Encourages reading	17	3
An attractive resource	15	2.6
A dynamic resource	12	2.1
Favours significant learning	11	1.9
Encourages research	8	1.4
Favours the capacity of synthesis	7	1.2
Transversality	7	1.2
Simple to carry out	4	0.7
Favours group work	3	0.5
Possibility of developing critical thinking	2	0.3
An accessible resource	1	0.2
Total	580	100

The analysis from the AQUAD 7 software offers up a series of results (Supplementary Material) which, *a priori*, are of interest as there is a total of 580 different open responses from the participants, from which 23 different variables can be extracted which are considered positive, such as: being an accessible resource which is, at the same time, entertaining and fun and fosters research and autonomous work on the part of the students (this is directly related with the fact that the educational action was, in this case, individual).

Three variables particularly stand out which the future teachers perceive to be positive concerning the use of comics in the teaching of history. On the one hand, the most repeated aspect was that it “encourages the understanding of conceptual contents” with 73 responses and 12.6% of the total answers. Secondly, it was considered to be very positive that comics are a “motivating” resource for students. This response was given 72 times with 12.4% of the answers. Thirdly, 69 responses (11.9%) highlighted the fact that comics are a resource which makes it possible to “encourage creativity.”

It is coherent that comics are considered to be a “motivating” resource given that it coincides with and reaffirms the good results of the quantitative items along the same lines. However, it is noteworthy that the most highly valued item regarding motivation was item 7 (The activity has motived me because we have used resources which are different to those normally used), whereas, in the open responses, it was highlighted that “it favours the understanding of historical contents,” with responses related with using “different resources in the classroom” being a long way behind, only appearing in 4.5% of the responses.

A good example can be found in the response of participant 108, who commented “It increases motivation, encourages creativity, helps children to understand the subject matter better as they use illustrations in the form of cartoon strips, without too much text and putting relevant information.” Furthermore, participant 178 considered that “on the other hand, comics encourage creativity, imagination, initiative and the go-getting spirit sought in so many other subjects.”

### Limitations and Difficulties When Using Comics in History Education

As far as item 12 of the questionnaire is concerned, the participants were required to indicate any negative aspects which they had perceived in the research (Table 12).

**Table 12:** Negative aspects regarding the use of comics as a resource for learning history.

Variable	Frequency	%
Preparation time	67	22.5
Difficulty in presenting/adapting contents	45	15.1
Time-consuming work	33	11.1
None	25	8.4
Lack of ICT training	19	6.4
Payment for applications	18	6
Perception of entertainment	17	5.7
Difficulty for students	12	4
Insufficient for learning historical contents	10	3.3
Lack of necessary (ICT) resources	9	3
Lack of training for teachers	9	3
Effort	8	2.7
Lack of creative and artistic skills	5	1.7
The elaboration of subsequent activities	5	1.7
Lack of motivation among students	4	1.3
Students do not like it	3	1
Perception of lack of learning	3	1
May cause disruptive behaviour in class	2	0.7
Necessary to know the specific language of comics	2	0.7
Intimidating for students due to its great amount of possibilities	1	0.3
The starting point	1	0.3
Total	298	100

In this case, the qualitative analysis of the open question (Supplementary Material) yielded a total of 298 different responses, representing almost half the number of negative responses compared to positive ones. Furthermore, 8.4% of them (a total of 25 responses) were merely to state that no great negative aspects had been found when applying the use of comics and creativity for teaching and learning history.

The trainee history teachers mainly pointed out as a negative aspect (22.5%) the “preparation time” required to create their own comic to represent a historical event or character. For example, participant 47 stated “One negative aspect could be the time it took to create. If a comic had to be made for each topic throughout the year, I do not think a teacher would have the time it would take.” The preparation for a comic also involves searching for historical information in order to make the data offered more rigorous. This coincides with the opinion of participant 176: “One of the negative aspects in creating comics is the time employed as it is necessary to plan the story and what you want to tell really well.”

Furthermore, 15.1% of the participants commented on the “difficulty in presenting and adapting historical contents to the comic format.” In this regard, participant 20 remarked: “The only negative aspect I can find is that the year group the comic is aimed at must be taken into consideration due to the fact that, as it is not a constant narration (which is what students are accustomed to), it can be difficult to follow and understand the story.” This aspect can even be reinforced with perceptions such as that of participant 55, who recalled that “perhaps the only drawback is that when representing certain facts, they can be interpreted as being closed and not continuous.”

Finally, the third most common negative aspect among the participants is that “it is a very time-consuming task,” with 33 responses (11.1% of the total). This is closely related with the lack of time to research, use sources, prepare the topic and, finally, to create their own comic. Thus, participant 123 commented: “It involves a lot of work because, first of all, you have to learn to use the application and then put the contents, search for pictures, etc. You have to be very careful about what you want to explain through the comic.” Even more than this, it was perceived that this negative aspect can be aggravated if the comic has to be devised and created by trainee primary teachers: “It is very time-consuming to create a comic, and even more so if you do so with primary schoolchildren” (participant 40).

## Discussion

The research presented in this paper is based on the prior assumption that surmised that the trainee primary teachers had never worked on history *via* a resource such as comics. Thus, the researchers wondered whether the future teachers would feel motivated teaching and learning history using comics as an educational resource.

In this regard, the prime objective posed in this research was to analyse the perceptions of trainee teachers regarding their degree of motivation when learning history through comics.

In order to achieve this aim, a specific educational programme on the use of comics in the teaching of the social sciences was designed, along with a mixed qualitative and quantitative questionnaire created *ad hoc* to evaluate the degree of motivation of the 221 participating trainee primary teachers from the University of Alicante. The results of the construct validity confirm that, methodologically speaking, the tool is highly reliable, as has been the case in other similar research projects on motivation in the teaching of the social sciences, such as that of Gómez-Carrasco et al. (2020).

When analysing the results, three different specific objectives were proposed. First of all, the different aspects of motivation perceived by the participants when using comics in social science classes have been researched (SO1). Here, it should be highlighted that the results concerning motivation have been extremely positive, with mean scores of above 4 out of a possible 5. Item 7 (The activity has motived me because we have used resources which are different to those normally used) stands out with the highest mean score of 4.6 and, furthermore, a positive result indicating that 90.5% of the participants were “in agreement” that working with educational resources which were different to those normally employed proves motivating in history classes.

This can basically be explained by the fact that the teaching of the social sciences, and history in particular, still maintains an extremely rigid and traditional structure (Estepa, 2017; Moreno-Vera and Alvén, 2020), in which the explanatory method (the masterclass) continues to be the most common approach when constructing new knowledge. The textbook is still the most commonly used educational resource in history classes, as pointed out by Vera et al. (2014), who, following an investigation of history teaching in Spain, concluded that more than 85% of teachers continued to employ textbooks as the only resource in the classroom. Thus, using resources other than the textbook in itself represents an element of motivation according to the future primary teachers.

This result is confirmed if the fact is taken into account that 84.1% of the trainee teachers feel motivated when they are responsible for their own learning. The participants in the study consider it to be a factor of motivation that it is the students who shoulder the responsibility of constructing knowledge (Powell and Kalina, 2009), putting into practice educational strategies which facilitate creativity (Cooper, 2018), historical research employing sources (Prats and Santacana, 2001; Ortega-Sánchez and Pagès, 2020), active learning methods (Gómez-Carrasco et al., 2019) and the use of narratives to learn history (Moreno-Vera, 2015).

Some of the results prove to be of particular interest, such as those of item 4, which have the lowest mean score of the study, thereby indicating that the trainee teachers do not attribute so much importance to motivation depending on the final mark that will be awarded.

SO2 was concerned with the analysis of the positive perceptions of the participants when using comics as a resource for the teaching of history. In this case, a wide variety of favourable opinions can be found among a total of 580 responses, among which those referring to the capacity of comics to “encourage understanding of historical contents” stand out, thus coinciding with prior research which has treated narrative resources as a fundamental element when learning history in primary and early-years education (Moreno-Vera, 2015), in which narration has been a significant element for researchers such as Egan (1994).

Indeed, as stated by González Gaxiola et al. (2020), one of the main reasons why teachers resort to graphic narratives (comics, graphic novels, etc.) is to facilitate understanding of abstract concepts among students. Without doubt, the narrative nature of comics with the combination of text and images enables a better understanding of the ideas and contents presented. Therefore, the visual aspect of this resource must be highlighted, as images have, in the words of Becerra Romero and Jorge Godoy (2014, p. 17), the “added value that they avoid pages and pages of descriptions and help to set the scene and contextualise the characters.” In this way, comics facilitate the understanding and visualisation of historical information (Saitua, 2018b; Ortega-Sánchez et al., 2019).

Furthermore, the results obtained *via* the open questions endorse the idea that comics are a tool with great potential in terms of motivating students (Altarriba, 2003; Gutiérrez, 2006; Saitua, 2018a). This perception is, doubtless, connected with the fact that they are entertaining, which, in the light of the participants’ responses, has passed from being considered prejudicial, as has traditionally been the case (Saitua, 2018a) and as can be observed in some of the responses regarding negative aspects, to being considered by the vast majority as a positive element which can attract the students’ interest and facilitate learning.

These positive results regarding the “motivation” which comics produce among students reinforce the encouraging data shown by the research on motivation *via* the analysis of the quantitative data. Last of all, the participants highlighted the capacity of comics as a resource to “foster students’ creativity,” thus coinciding with Orlich et al. (1994) and Baur (1978), who stated that, in order to achieve creative thinking, practices which foster it must be worked on in the classroom.

SO3 focused on the qualitative analysis of the possible difficulties, limitations and negative aspects of the use of comics in history classes. In this regard, of a total of 298 responses, the most noteworthy were focused on formal aspects.

Without doubt, introducing comics into the classroom, be it *via* pre-existing editions or original creations, has a series of limitations or negative aspects which should be taken into account, such as the preparation time required (mentioned by 22.5% of the participants) and, in many cases, the work required, implying the preparation of materials, the use of sources and historical evidence (Seixas and Morton, 2013), research (Prats and Santacana, 2001) and, last of all, the creation of the comic itself.

Likewise, another of the drawbacks or objections mentioned by the participants relates to the difficulty of presenting or adapting historical contents to the format of a comic (15.1% of the total). In this regard, it must be highlighted that this requires a significant capacity for synthesis and organisation of the structure of the comic. However, as stated by Becerra Romero and Jorge Godoy (2014), it should be taken into account that comics are not history books, but rather a resource or complementary material to assist students in approaching a specific topic or subject.

In conclusion, therefore, it can be affirmed that the trainee primary education teachers consider the use of comics to be a motivating resource for the teaching and learning of history, particularly because it makes it possible to work with resources other than the textbook, something that also happen in geography, according to authors like Morote et al. (2021). Furthermore, they consider the use of comics to be extremely useful for their future careers. In addition, they highlight as a positive aspect the fact that comics are capable of facilitating the understanding of historical contents, fostering creativity and motivating their future students. As far as negative aspects are concerned, the participants highlight the excessive time and difficulty implied by creating an original comic in class and the difficulty of correctly adapting historical contents to the format of a comic.

## Data Availability Statement

The original contributions presented in the study are included in the article/Supplementary Material; further inquiries can be directed to the corresponding author.

## Ethics Statement

The studies involving human participants were reviewed and approved by Comité de ética de la Universidad de Murcia. The patients/participants provided their written informed consent to participate in this study.

## Author Contributions

JM-V: materials and methods, quantitative analysis and results, and discussion. SP-L: instrument design, data collection, educational programme design, qualitative results, and discussion. RB-M: introduction, references, data collection, and discussion. All authors contributed to the article and approved the submitted version.

## Funding

This research is part of the project “Accept the challenge! Gamification in online higher education” (2020-1-PL01-KA226-HE-096034) funded by the European Union ERASMUS+ KA2 programme Cooperation and innovation for good practices.

## Conflict of Interest

The authors declare that the research was conducted in the absence of any commercial or financial relationships that could be construed as a potential conflict of interest.

## Publisher’s Note

All claims expressed in this article are solely those of the authors and do not necessarily represent those of their affiliated organizations, or those of the publisher, the editors and the reviewers. Any product that may be evaluated in this article, or claim that may be made by its manufacturer, is not guaranteed or endorsed by the publisher.
