# Supplementary material for: By Toutatis! Trainee Teachers’ Motivation When Using Comics to Learn History
Source: Front Psychol. 2021 Oct 27;12:778792. doi: 10.3389/fpsyg.2021.778792 (PMC8578684; doi:10.3389/fpsyg.2021.778792)
Supplement: Supplementary file 1 [file Table_1.DOCX]

Supplementary Material

# Supplementary Figures and Tables

**RESEARH INSTRUMENT (QUESTIONNAIRE, LIKERT SCALE 1-5)**

| **Responde a las siguientes afirmaciones:**  1 Totalmente en desacuerdo  2 algo en desacuerdo  3 Ni de acuerdo ni en desacuerdo  4 Algo de acuerdo  5 Totalmente de acuerdo | | **Nunca**  **Totalmente en desacuerdo** | **Raramente**  **Algo en desacuerdo** | **Ocasionalmente**  **Ni de acuerdo ni en desacuerdo** | **Frecuentemente**  **Algo de acuerdo** | **Muy frecuentemente**  **Totalmente de acuerdo** |
| --- | --- | --- | --- | --- | --- | --- |
| **BLOQUE I. El cómic como recurso didáctico** | | | | | | |
| 1 | La forma de presentar y trabajar la actividad me ha motivado para conocer más sobre la historia. | 1 | 2 | 3 | 4 | 5 |
| 2 | La forma de presentar y trabajar la actividad me ha motivado para conocer más sobre el uso del cómic como recurso para enseñar-aprender historia. | 1 | 2 | 3 | 4 | 5 |
| 3 | La actividad ha mejorado mi motivación para esforzarme más en la asignatura. | 1 | 2 | 3 | 4 | 5 |
| 4 | La actividad ha mejorado mi motivación para conseguir mejores notas. | 1 | 2 | 3 | 4 | 5 |
| 5 | La actividad me ha motivado porque permitía aportar mis conocimientos. | 1 | 2 | 3 | 4 | 5 |
| 6 | La actividad me ha motivado porque permitía aportar mi capacidad creativa. | 1 | 2 | 3 | 4 | 5 |
| 7 | La actividad me ha motivado porque hemos utilizados recursos diferentes a los habituales. | 1 | 2 | 3 | 4 | 5 |
| 8 | La actividad me ha motivado porque he podido ser protagonista de mi propio aprendizaje. | 1 | 2 | 3 | 4 | 5 |
| 9 | La actividad me ha motivado porque me ha enseñado a diseñar mis propios contenidos y actividades a través del comic. | 1 | 2 | 3 | 4 | 5 |
| 10 | La actividad me ha motivado porque la considero útil para mi futuro profesional. | 1 | 2 | 3 | 4 | 5 |

**11. Señala y explica los aspectos positivos de la elaboración de cómics para la enseñanza-aprendizaje de la Historia en Educación Primaria**

**12.Señala y explica los aspectos negativos de la elaboración de cómics para la enseñanza-aprendizaje de la Historia en Educación Primaria**

**2 Supplementary Figures and Tables**

**RELIABILITY: CRONBACH’S ALPHA**

| Alfa de Cronbach | N de elementos |
| --- | --- |
| ,902 | 10 |

**3** **Supplementary Figures and Tables**

**GUTTMAN’S – SPLIT-HALF**

| Correlación entre formularios | | ,778 |
| --- | --- | --- |
| Coeficiente de Spearman-Brown | Longitud igual | ,875 |
|  | Longitud desigual | ,875 |
| Coeficiente de dos mitades de Guttman | | ,873 |
| a. Los elementos son: VAR00001, VAR00002, VAR00003, VAR00004, VAR00005. | | |
| b. Los elementos son: VAR00006, VAR00007, VAR00008, VAR00009, VAR00010. | | |

**4** **Supplementary Figures and Tables**

**FREIDMANN’S CHI-SQUARE**

|  | VAR00001 | VAR00002 | VAR00003 | VAR00004 | VAR00005 | VAR00006 | VAR00007 | VAR00008 | VAR00009 | VAR00010 |  |
| --- | --- | --- | --- | --- | --- | --- | --- | --- | --- | --- | --- |
| Chi-cuadrado | 223,140^a^ | 249,475^a^ | 101,914^b^ | 69,299^b^ | 169,837^a^ | 273,683^a^ | 264,629^b^ | 201,557^a^ | 147,814^b^ | 333,910^a^ |  |
| gl | 4 | 4 | 3 | 3 | 4 | 4 | 3 | 4 | 3 | 4 |  |
| Sig. asintótica | ,000 | ,000 | ,000 | ,000 | ,000 | ,000 | ,000 | ,000 | ,000 | ,000 |  |
| a. 0 casillas (0,0%) han esperado frecuencias menores que 5. La frecuencia mínima de casilla esperada es 44,2. | | | | | | | | | | | |
| b. 0 casillas (0,0%) han esperado frecuencias menores que 5. La frecuencia mínima de casilla esperada es 55,3. | | | | | | | | | | | |

**5** **Supplementary Figures and Tables**

**QUANTITATIVE ANALYSIS**

**AVERAGE AND STANDARD DEVIATION**

|  | | VAR00001 | VAR00002 | VAR00003 | VAR00004 | VAR00005 | VAR00006 | VAR00007 | VAR00008 | VAR00009 | VAR00010 |
| --- | --- | --- | --- | --- | --- | --- | --- | --- | --- | --- | --- |
| N | Válido | 221 | 221 | 221 | 221 | 221 | 221 | 221 | 221 | 221 | 221 |
|  | Perdidos | 0 | 0 | 0 | 0 | 0 | 0 | 0 | 0 | 0 | 0 |
| Average | | 4,3575 | 4,4118 | 4,1810 | 4,0452 | 4,2127 | 4,4570 | 4,6018 | 4,3032 | 4,3891 | 4,5475 |
| Mediana | | 5,0000 | 5,0000 | 4,0000 | 4,0000 | 4,0000 | 5,0000 | 5,0000 | 4,0000 | 5,0000 | 5,0000 |
| Standar Deviation | | ,79418 | ,76137 | ,77095 | ,82997 | ,91205 | ,75931 | ,69727 | ,81656 | ,75237 | ,71589 |

**6** **Supplementary Figures and Tables**

**STATISTICAL ANALYSIS BY ITEMS**

| **VAR00001** | | | | | |
| --- | --- | --- | --- | --- | --- |
|  | | Frecuencia | Porcentaje | Porcentaje válido | Porcentaje acumulado |
| Válido | 1,00 | 2 | ,9 | ,9 | ,9 |
|  | 2,00 | 1 | ,5 | ,5 | 1,4 |
|  | 3,00 | 29 | 13,1 | 13,1 | 14,5 |
|  | 4,00 | 73 | 33,0 | 33,0 | 47,5 |
|  | 5,00 | 116 | 52,5 | 52,5 | 100,0 |
|  | Total | 221 | 100,0 | 100,0 |  |

| **VAR00002** | | | | | |
| --- | --- | --- | --- | --- | --- |
|  | | Frecuencia | Porcentaje | Porcentaje válido | Porcentaje acumulado |
| Válido | 1,00 | 1 | ,5 | ,5 | ,5 |
|  | 2,00 | 4 | 1,8 | 1,8 | 2,3 |
|  | 3,00 | 19 | 8,6 | 8,6 | 10,9 |
|  | 4,00 | 76 | 34,4 | 34,4 | 45,2 |
|  | 5,00 | 121 | 54,8 | 54,8 | 100,0 |
|  | Total | 221 | 100,0 | 100,0 |  |

| **VAR00003** | | | | | |
| --- | --- | --- | --- | --- | --- |
|  | | Frecuencia | Porcentaje | Porcentaje válido | Porcentaje acumulado |
| Válido | 2,00 | 5 | 2,3 | 2,3 | 2,3 |
|  | 3,00 | 34 | 15,4 | 15,4 | 17,6 |
|  | 4,00 | 98 | 44,3 | 44,3 | 62,0 |
|  | 5,00 | 84 | 38,0 | 38,0 | 100,0 |
|  | Total | 221 | 100,0 | 100,0 |  |

| **VAR00004** | | | | | |
| --- | --- | --- | --- | --- | --- |
|  | | Frecuencia | Porcentaje | Porcentaje válido | Porcentaje acumulado |
| Válido | 2,00 | 6 | 2,7 | 2,7 | 2,7 |
|  | 3,00 | 53 | 24,0 | 24,0 | 26,7 |
|  | 4,00 | 87 | 39,4 | 39,4 | 66,1 |
|  | 5,00 | 75 | 33,9 | 33,9 | 100,0 |
|  | Total | 221 | 100,0 | 100,0 |  |

| **VAR00005** | | | | | |
| --- | --- | --- | --- | --- | --- |
|  | | Frecuencia | Porcentaje | Porcentaje válido | Porcentaje acumulado |
| Válido | 1,00 | 1 | ,5 | ,5 | ,5 |
|  | 2,00 | 11 | 5,0 | 5,0 | 5,4 |
|  | 3,00 | 34 | 15,4 | 15,4 | 20,8 |
|  | 4,00 | 69 | 31,2 | 31,2 | 52,0 |
|  | 5,00 | 106 | 48,0 | 48,0 | 100,0 |
|  | Total | 221 | 100,0 | 100,0 |  |

| **VAR00006** | | | | | |
| --- | --- | --- | --- | --- | --- |
|  | | Frecuencia | Porcentaje | Porcentaje válido | Porcentaje acumulado |
| Válido | 1,00 | 1 | ,5 | ,5 | ,5 |
|  | 2,00 | 4 | 1,8 | 1,8 | 2,3 |
|  | 3,00 | 18 | 8,1 | 8,1 | 10,4 |
|  | 4,00 | 68 | 30,8 | 30,8 | 41,2 |
|  | 5,00 | 130 | 58,8 | 58,8 | 100,0 |
|  | Total | 221 | 100,0 | 100,0 |  |

| **VAR00007** | | | | | |
| --- | --- | --- | --- | --- | --- |
|  | | Frecuencia | Porcentaje | Porcentaje válido | Porcentaje acumulado |
| Válido | 2,00 | 3 | 1,4 | 1,4 | 1,4 |
|  | 3,00 | 18 | 8,1 | 8,1 | 9,5 |
|  | 4,00 | 43 | 19,5 | 19,5 | 29,0 |
|  | 5,00 | 157 | 71,0 | 71,0 | 100,0 |
|  | Total | 221 | 100,0 | 100,0 |  |

| **VAR00008** | | | | | |
| --- | --- | --- | --- | --- | --- |
|  | | Frecuencia | Porcentaje | Porcentaje válido | Porcentaje acumulado |
| Válido | 1,00 | 1 | ,5 | ,5 | ,5 |
|  | 2,00 | 5 | 2,3 | 2,3 | 2,7 |
|  | 3,00 | 29 | 13,1 | 13,1 | 15,8 |
|  | 4,00 | 77 | 34,8 | 34,8 | 50,7 |
|  | 5,00 | 109 | 49,3 | 49,3 | 100,0 |
|  | Total | 221 | 100,0 | 100,0 |  |

| **VAR00009** | | | | | |
| --- | --- | --- | --- | --- | --- |
|  | | Frecuencia | Porcentaje | Porcentaje válido | Porcentaje acumulado |
| Válido | 2,00 | 6 | 2,7 | 2,7 | 2,7 |
|  | 3,00 | 18 | 8,1 | 8,1 | 10,9 |
|  | 4,00 | 81 | 36,7 | 36,7 | 47,5 |
|  | 5,00 | 116 | 52,5 | 52,5 | 100,0 |
|  | Total | 221 | 100,0 | 100,0 |  |

| **VAR00010** | | | | | |
| --- | --- | --- | --- | --- | --- |
|  | | Frecuencia | Porcentaje | Porcentaje válido | Porcentaje acumulado |
| Válido | 1,00 | 1 | ,5 | ,5 | ,5 |
|  | 2,00 | 1 | ,5 | ,5 | ,9 |
|  | 3,00 | 20 | 9,0 | 9,0 | 10,0 |
|  | 4,00 | 53 | 24,0 | 24,0 | 33,9 |
|  | 5,00 | 146 | 66,1 | 66,1 | 100,0 |
|  | Total | 221 | 100,0 | 100,0 |  |

**7** **Supplementary Figures and Tables**

**QUALITATIVE ANALYSIS**

**ÍTEM 11 POSITIVE**

|  |  | FA | %FA | Narrativas |
| --- | --- | --- | --- | --- |
| 16 | Favorece la  comprensión  de contenidos | 73 | 12.6 | P.003 - Permite que el alumnado aprenda contenidos históricos de forma divertida y no meramente memorizando.  P.018- Por otra parte me parece una buena forma de introducir nuevos contenidos y de tratar el temario de Historia que puede ser sea más complicado para ellos y ellas. El cómic nos permite trabajar contenidos históricos lejanos a la realidad del alumnado a través de un recurso cercano a ellos y ellas y que incluso puede ser lúdico  P.025- Primeramente, es importante recalcar que trabajar contenidos a partir de un relato es mucho más motivante para el alumnado y les ayuda a tener un papel más activo en su propio aprendizaje. Además, trabajar con dibujos, viñetas y fragmentos de texto les acerca más al pasado histórico que muchas veces se ve alejado y sin referencias.  P.074 – Nos permite tratar contenidos de tipo histórico a través de historietas, facilitando la comprensión de los mismos y favoreciendo la motivación de los alumnos.  P.078- es una manera de trabajar los diferentes contenidos de una manera que el niño los interiorice de una manera más sencilla, práctica y significativa.  P.093-Los cómics ayudan a que los alumnos/as comprendan el contenido de las asignaturas de una forma más visual y con un vocabulario sencillo que ayude a su comprensión y a la interiorización de los conocimientos de manera significativa  P.143- Los cómics me parecen que pueden resultar atractivos a los niños/as a la hora de aprender historia, ya que están acostumbrados a un aprendizaje memorístico y con este recurso pueden cambiar su perspectiva. De forma que puedan crear sus propios cómic e historia a partir de los contenidos dados en clase y consoliden los contenidos de forma significativo.  P.196- Aumenta la motivación del alumnado, mejora el desarrollo de la creatividad y favorece a la interiorización de los contenidos teóricos.  P.205- Del mismo modo, huir de los libros de texto presentando esta alternativa, favorece que se adquieran conocimientos por otra vía que no sea la memorística, ya que da pie a despertar la creatividad, la imaginación y a situarse en una posición mucho más activa.  P.210- Desarrolla la creatividad, se afianzan los conocimientos más rápido y mejor.  P.212- Permite al alumnado comprender mejor los hechos, ya que desarrolla empatía por los personajes presentados. |
| 1 | Motivación | 72 | 12.4 | P.006- Uno de los principales aspectos positivos de esta forma de trabajar es la capacidad de motivación del alumnado. El hecho de elegir este recurso tan cercano a ellos i que tanto les gusta hace que su disposición ya sea buena  P.016- Mediante el cómic se hace la enseñanza de la historia mucho más motivadora a la vez que interesante para gran parte del alumnado.  P.102- Entiendo que, por su aspecto, es útil para trabajar la historia en Educación Primaria, ya que al alumnado le puede resultar mas interesante y motivador.  P.103- Los alumnos pueden estar mucho más motivados a la hora de aprender contenidos nuevos  P.108- Aumenta la motivación, fomenta la creatividad, ayuda a que los niños comprendan mejor el temario ya que utilizan ilustraciones en forma de viñetas, sin abusar del texto y poniendo la información relevante.  P.125- Es un elemento ya existente de hace mucho tiempo, pero puede ser muy innovador si lo planteamos de forma adecuada en el aula  P.164-Motivador y diferente a todo lo realizado con anterioridad. Como futura docente es una herramienta que pondré en pràctica con mi futuro alumnado.  P.196- Aumenta la motivación del alumnado, mejora el desarrollo de la creatividad y favorece a la interiorización de los contenidos teóricos.  P.198- Motiva al alumnado puesto que les interesa mucho más leer un cómic con diálogos que un texto larguísimo que te cuente un hecho histórico. |
| 12 | Fomenta la creatividad | 69 | 11.9 | P.019- Mejoran la creatividad y hacen que los niños sean los que crean su propio aprendizaje  P.041- Esta actividad es muy interesante ya que puedes desarrollar tu creatividad además nos obliga a aprender de manera autónoma  P.044-Una de las ventajas de la elaboración de cómics para la enseñanza-aprendizaje de la Historia es que es un recurso muy creativo que puede resultar muy motivador para el alumnado.  P.099- Desarrollo de habilidades artísticas como la imaginación y la creatividad.  P.118-Hay varios aspectos positivos de la elaboración de cómics para la EA de la Historia en Educación Primaria como son por ejemplo el fomento de la creatividad, mayor motivación del alumnado de cara a las actividades, aprender contenidos de una forma lúdica etc  P.138.- Te permite desarrollar la creatividad a partir de unos determinados conocimientos, es decir, aquello que conocemos bastante como las TIC, hemos podido emplear dichos saberes de una forma bastante creativa.  P.146- desarrolla la creatividad y la imaginación. De esta manera, el alumnado adquirirá nuevos conocimientos de manera lúdica y creativa.  P.160-Para mí creo que es una manera original de utilizar la creatividad y la imaginación para crear un recurso interesante y diferente al utilizado siempre para explicar historia.  P.178- Por otra parte, fomenta la creatividad, imaginación y el espíritu de iniciativa y emprendedor que tanto se busca en muchas otras asignaturas.  P.196- Aumenta la motivación del alumnado, mejora el desarrollo de la creatividad y favorece a la interiorización de los contenidos teóricos.  P.205-Del mismo modo, huir de los libros de texto presentando esta alternativa, favorece que se adquieran conocimientos por otra vía que no sea la memorística, ya que da pie a despertar la creatividad, la imaginación y a situarse en una posición mucho más activa.  P.210- Desarrolla la creatividad, se afianzan los conocimientos más rápido y mejor.  P.219- Mediante la elaboración de un cómic, el alumnado puede expresarse y plasmar sus ideas de una forma más creativa, lo que favorece la retención de conocimientos y la adquisición de unos nuevos. |
| 6 | Recurso visual facilita la comprensión | 46 | 7.9 |  |
| 2 | Lúdico | 45 | 7.7 |  |
| 10 | Recurso innovador/ innovación | 37 | 6.4 |  |
| 7 | Permite adaptar contenidos | 28 | 4.8 |  |
| 9 | Favorece el aprendizaje autónomo y activo en su aprendizaje | 28 | 4.8 |  |
| 24 | Permite trabajar competencias bàsicas | 27 | 4.6 |  |
| 3 | Permite aprender de manera diferente | 26 | 4.5 |  |
| 17 | Favorece trabajar competencias asociadas al desarrollo del pensamiento histórico | 23 | 4 |  |
| 13 | Despierta la curiosidad/interés/atención del alumnado | 19 | 3.3 |  |
| 14 | Fomenta la lectura | 17 | 3 |  |
| 11 | Recurso atractivo | 15 | 2.6 |  |
| 31 | Recurso dinámico | 12 | 2.1 |  |
| 4 | Favorece el aprendizaje significativo | 11 | 1.9 |  |
| 30 | Fomenta la indagación | 8 | 1.4 |  |
| 26 | Favorece la capacidad de síntesis, | 7 | 1.2 |  |
| 32 | Transvesalidad | 7 | 1.2 |  |
| 29 | Sencillez para realizar | 4 | 0.7 |  |
| 22 | Favorece rabajo colectivo | 3 | 0.5 |  |
| 15 | Posibilidad de desarrollar un pensamiento crítico | 2 | 0.3 |  |
| 19 | Recurso accesible | 1 | 0.2 |  |
|  |  | 580 | 100 |  |

**8** **Supplementary Figures and Tables**

**QUALITATIVE ANALYSIS**

**ÍTEM 12 NEGATIVE**

|  |  | FA | %FA |  |
| --- | --- | --- | --- | --- |
| 2 | Tiempo de preparación | 67 | 22.5 | P.009- Es un trabajo muy complicado que lleva su tiempo para hacer[...]  P.015- los inconvenientes sería el tiempo de preparación y la no preparación del professora  P.027-Un aspecto negativo podria ser el tiempo de elaboración.  P.034-Se necessita bastante tiempo para preparar el còmic.  P.047- Un aspecto negativo podría ser el tiempo de elaboración. Si se quisiera diseñar cómics para cada tema del curso, creo que un profesor no dispondría del tiempo real que se necesita.  P.060- Requiere una cantidad de tiempo bastante importante (al menos para mi) y te quita tiempo para preparar otras actividades que podrían ser igual o más significativas.  P.114- El tiempo que se necesita y la organización.  P.176- Uno de los aspectos negativos que tiene la elaboración de cómics es el tiempo empleado a la hora de realizarlo ya que se se tiene que pensar muy bien la historia y lo que pretendes contar.  P.187- La necesidad de mucho tiempo para realizar el cómic y la unión con las actividades posteriores |
| 11 | Difultad para presentar/adaptar contenidos | 45 | 15.1 | 20-El único aspecto negativo que puedo encontrar es que se debería tenir muy en cuenta el curso al que va dirigido porque al no ser una narración constante (que es a lo que el alumnado està acostumbrado) puede resultar difícil a la hora de seguir la historia y entenderla.  P.023-Tan solo puedo mencionar un aspecto negativo, es difícil seleccionar la información histórica que se introduce en el còmic.  P.030-Puede resultar complicada su comprensión si no se realiza un buen uso o se adecuan los diferentes elementos que se han de tener en cuenta (edat, tema,..)  P.036-Puede resultar insuficiente para profundizar en las diferentes època històricas.  P.055- Tal vez el único inconveniente que haya es que al representar en ellos ciertos hechos, pueden interpretarse como cerrados y no continuos.  P.087- Considero que los temas de historia son bastante extensos por lo tanto, no creo que con un comic se pueda explicar todo.  P.097- Es un formato caracterizado por la brevedad del texto escrito y por tanto se hace ardua la tarea de enseñanza de ciertos contenidos históricos.  P.108- Por poner algún aspecto negativo, pienso que los niños puede ser que no lleguen a entender ese concepto de aprender historia mediante un cómic y cabe la posibilidad de que se despisten con las ilustraciones.  P.133- La parte negativa puede ser que quizás no cabe tanta información como nos gustaría trasmitir al alumnado.  P.140- Visualizar a través del comic la historia con todas sus características, me parece complicado y un poco difícil de representar.  P.143- A pesar de la motivación, el interés y la creatividad que pueda desarrollar el alumnado, me parece un poco complicado sintetizar los contenidos más importantes que quieran transmitir mediante unas imágenes. |
| 5 | Trabajo laborioso | 33 | 11.1 | P.026 [...]También otro aspecto (negativo) es el trabajo que comporta para el docente. [...]  P.040- Es muy laborioso realitzar un còmic y más si lo haces con alumnos de primària.  P.050- La elaboración de cómics es un trabajo muy laborioso si se quiere hacer de una manera atractiva e interesante.  P.054- Conlleva bastante trabajo por parte del docente, por lo que puede ser complicado diseñar uno para cada contenido.  P.081- Creo que la única parte negativa es el trabajo que conlleva crear el comic, ya que por lo demás me parece una muy buena herramienta.  P.123-Conlleva bastante trabajo porque primero hay que aprender a manejarse en la aplicación y luego poner los contenidos, buscando imagenes y demás. Hay que ser cuidadoso a la hora de saber que se quiere explicar mediante el còmic  P.220- Puede ser algo laborioso y poco fructífero si no se organiza de una forma detallada, por lo que hay que tener un planning previo, con una guía y fomentar la puesta en grupo de dudas y preguntas sobre el cómic, ya que sin ello se puede llegar a la confusión y a que la actividad no sea del todo rentable en cuanto al fin que queremos obtener con esta elaboración. |
| 12 | Niguna | 25 | 8.4 |  |
| 9 | Falta de formación TIC | 19 | 6.4 |  |
| 13 | Pago de aplicacions | 18 | 6 |  |
| 15 | Percepción lúdica | 17 | 5.7 |  |
| 8 | Dificultad para el alumnado | 12 | 4 |  |
| 17 | Insuficente para aprender contenidos históricos | 10 | 3.3 |  |
| 1 | Falta de recursos (TIC) necesarios | 9 | 3 |  |
| 14 | Falta de formación del profesorado | 9 | 3 |  |
| 3 | Esfuerzo | 8 | 2.7 |  |
| 18 | Falta de capacidad creativa y artística | 5 | 1.7 |  |
| 19 | La elabroación de actividades posteriores | 5 | 1.7 |  |
| 4 | Falta de motivación del alumnado | 4 | 1.3 |  |
| 7 | No guste al alumnado | 3 | 1 |  |
| 16 | Percepción de que no se aprende | 3 | 1 |  |
| 20 | Puede provocar conductes disruptivas en el aula | 2 | 0.7 |  |
| 21 | Intimidar al alumnado por su gran cantidad de posibilidades | 1 | 0.3 |  |
| 22 | Se ha de concoer el lenguaje propio deel cómic | 2 | 0.7 |  |
| 23 | El punto departida | 1 | 0.3 |  |
|  |  | 298 | 100 |  |
